# Nucleotide diversity inflation as a genome-wide response to experimental lifespan extension in *Drosophila melanogaster*

**DOI:** 10.1186/s12864-017-3485-0

**Published:** 2017-01-14

**Authors:** Pawel Michalak, Lin Kang, Pernille M. Sarup, Mads F. Schou, Volker Loeschcke

**Affiliations:** 1 Biocomplexity Institute, Virginia Tech, 1015 Life Science Circle, Blacksburg, VA 24061 USA; 2 Department of Bioscience, Aarhus University, Ny Munkegade 114-116, Aarhus, DK-8000 Denmark

## Abstract

**Background:**

Evolutionary theory predicts that antagonistically selected alleles, such as those with divergent pleiotropic effects in early and late life, may often reach intermediate population frequencies due to balancing selection, an elusive process when sought out empirically. Alternatively, genetic diversity may increase as a result of positive frequency-dependent selection and genetic purging in bottlenecked populations.

**Results:**

While experimental evolution systems with directional phenotypic selection typically result in at least local heterozygosity loss, we report that selection for increased lifespan in *Drosophila melanogaster* leads to an extensive genome-wide increase of nucleotide diversity in the selected lines compared to replicate control lines, pronounced in regions with no or low recombination, such as chromosome 4 and centromere neighborhoods. These changes, particularly in coding sequences, are most consistent with the operation of balancing selection and the antagonistic pleiotropy theory of aging and life history traits that tend to be intercorrelated. Genes involved in antioxidant defenses, along with multiple lncRNAs, were among those most affected by balancing selection. Despite the overwhelming genetic diversification and the paucity of selective sweep regions, two genes with functions important for central nervous system and memory, *Ptp10D* and *Ank2*, evolved under positive selection in the longevity lines.

**Conclusions:**

Overall, the ‘evolve-and-resequence’ experimental approach proves successful in providing unique insights into the complex evolutionary dynamics of genomic regions responsible for longevity.

**Electronic supplementary material:**

The online version of this article (doi:10.1186/s12864-017-3485-0) contains supplementary material, which is available to authorized users.

## Background

Developing the evolutionary theory of aging has been a key endeavor since the very beginning of the modern evolutionary synthesis [1, 2]. Two major non-mutually exclusive models of how aging can originate and evolve have been formulated. Both of them rest on the fact that natural selection is weak at old age due to small cohort size and declining contributions to reproduction. One stems from Medawar's ideas [3] that drift and mutation accumulation results in the loss of late-acting beneficial alleles or the emergence of late-acting deleterious alleles [2, 4]. Another is based on Williams’s model of pleiotropy [5] in which aging evolves as a consequence of pleiotropic effects of some genes that are beneficial early in life and then harmful at later ages. A corollary to the antagonistic pleiotropy theory of aging is that late-life selection operating on genes with pleiotropic effects will lead to the establishment and maintenance of genetic polymorphism, effectively becoming balancing selection [6]. However, the conditions for balancing selection due to antagonistic pleiotropy are fairly restrictive compared with balancing selection due to selective pressures varying in time and space [7–9].

While genomic studies now suggest balancing selection might be relatively common, to date most evidence for it remains indirect. For example, the relative levels of the effects of artificial selection on the population mean and inbreeding depression for the selected trait can separate variation maintained largely by mutation-selection balance and variation from a contribution from alleles at intermediate frequencies (tantamount to balancing selection) [10]. Intermediate allele frequencies played a key role in a selection experiment on female fecundity in *Drosophila melanogaster* [11], similar to selection on several traits in the monkeyflower *Mimulus guttatus* [12, 13]. Balancing selection could also account for the genetic variance in viability linked to the small fourth chromosome of *D. melanogaster* [14, 15], and polymorphism in numerous sites across *Drosophila* genomes seem to be maintained by balancing selection due to seasonal oscillations in climate [16].

Although through a different mechanism, the mutation accumulation theory of aging also predicts that polymorphism will be increased among genes with age-specific effects. As long as late-expressed alleles have no or very little differential effect on fitness, and the older the age the less fitness effects are indeed expected, such alleles will be subject to mostly neutral evolution, notwithstanding their discernible phenotypes [6]. Other processes, such as positive frequency-dependent selection [17] and purging selection against homozygotes for deleterious alleles affects [18] may also boost genetic polymorphism, especially when a reduction in population size is involved.

To test the prediction that selection for longevity increases genetic diversity, here we survey genome-wide patterns of nucleotide polymorphism in *Drosophila melanogaster* experimentally selected for increased lifespan [19–21]. Unsurprisingly, *Drosophila* have been a historically important system for investigating the genetic underpinnings of longevity [22, 23], and recently have become an even more attractive model owing to their tractable, relatively small genomes. In addition to dissecting the genetic basis of longevity, *Drosophila* have been indispensable in investigating the physiology of aging [24–26].

Experimental evolution employs well-established selection protocols to enforce phenotypic divergence, which coupled with the genome-wide analysis (‘evolve-and-resequence’) may narrow down the candidate target regions under selection, and provide a powerful alternative to genome-wide association studies (GWASs) and linkage mapping experiments as strategies to link genotype with phenotype [27–32]. A major advantage of experimental evolution compared to other evolutionary approaches is its ability to distinguish between stochastic and deterministic effects based on parallel replicates under controlled conditions. Another advantage of experimental evolution is that selection for life history traits, such as longevity, under a controlled laboratory environment with ample food, reduced competition and other antagonistic interactions is less likely to be affected by the constraints posed by trade-offs in suboptimal environments under natural conditions [33, 34].

## Results

### Variant discovery analysis

**Fig. 1:**
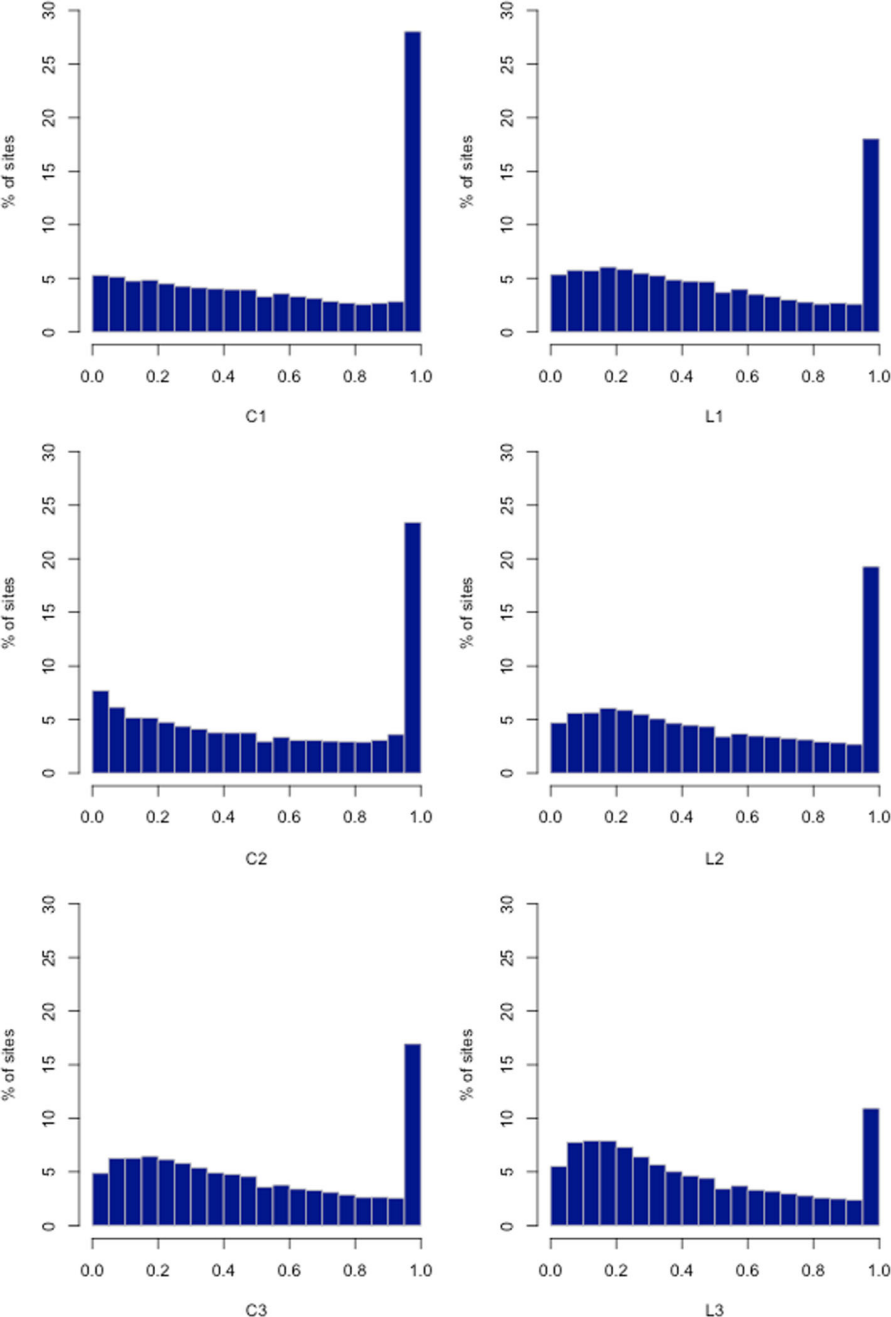
Site frequency spectra (SFSs) in three control (C1-C3) and three longevity (L1-L3) *Drosophila melanogaster* lines

Three longevity lines after 48 generations of selection and three parallel control lines (standard laboratory conditions without selection) were sequenced using pooled genomes per line (Pool-seq). We found a total of 1,497,961 polymorphic sites, 1,212,878 of which were heterozygous in all longevity lines and 1,050,542 were heterozygous in all control lines. A total of 192,558 SNPs were homozygous in all longevity lines while being heterozygous in at least one of the control lines, and, conversely, 305,601 SNPs were fixed in all control lines while heterozygous in at least one of the longevity lines. Only 169 SNPs were fixed in all selection lines with an alternative allele fixed in all control lines. Average SNP-based F_ST_ estimates were the lowest between the longevity lines (0.082) and the highest in control-longevity pairwise comparisons (0.124), compared with those between control lines (0.104). The inspection of site frequency spectra (SFSs) indicates that there were substantially more alleles with intermediate frequencies in the longevity lines than control lines (Fig. 1).

### Heterozygosity and Tajima’s D analysis

**Fig. 2:**
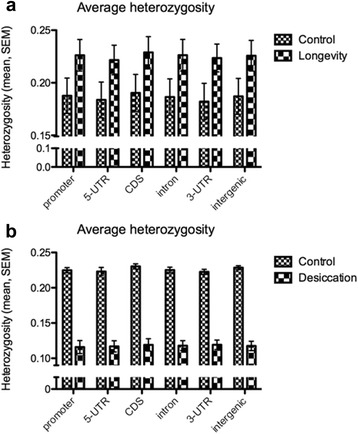
Average heterozygosity (±SEM) across various genomic regions in (**a**) control and longevity *D. melanogaster* lines, compared with (**b**) an experimental evolution system selected for desiccation resistance [42]

**Fig. 3:**
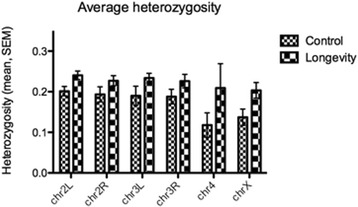
Average heterozygosity (±SEM) across chromosome arms

**Fig. 4:**
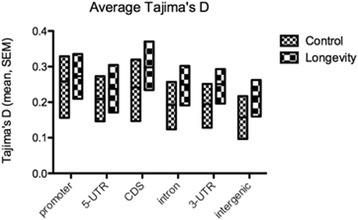
Profiles of average Tajima’s D values (±SEM) across various genomic regions

**Fig. 5:**
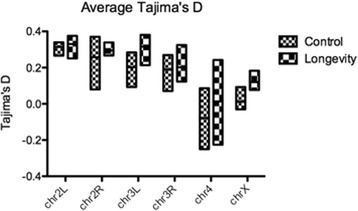
Profiles of average Tajima’s D values (±SEM) across chromosomes

Overall, mean heterozygosity was 21% higher in longevity lines relative to control lines, across all genomic regions, including coding sequences (Fig. 2a). The highest heterozygosity increase (~77%) was recorded for chromosome 4 and the lowest (17%) for chromosome arm 2R (Fig. 3). The genome-wide profiles of Tajima’s D values produced a similar result, with significant differences (Mann–Whitney test, *P* < 0.001) between control and longevity lines across all genomic regions (except the promoter region, Fig. 4) and across all chromosomes (Fig. 5), implying an extensive increase of nucleotide diversity in the selected lines. The highest Tajima’s D values were recorded in CDSs and chromosome 2L of the longevity lines (Fig. 5). The difference in heterozygosity and Tajima’s D between control and longevity lines was most pronounced in the centromere regions (2 Mb each) of chromosomes 2 and 3 (47% heterozygosity increase in longevity lines compared with the 19% increase in non-centromere regions), and the ~71 Kb *yellow-achaete-scute* complex near the tip of chromosome X (2,281% increase compared with 47% increase along the rest of the chromosome X) (Table 1).

**Table 1:** Mean heterozygosity and Tajima’s D estimates in regions expected to differ in the levels of recombination, including 2Mb neighborhoods of autosome centromeres and the ~71 Kb *yellow-achaete-scute* complex (Y-AS-C) on chromosome X

Heterozygosity				
	Centromeres	Non-centromeres	Y-AS-C	Non-Y-AS-C
Longevity lines	0.244	0.231	0.381	0.203
Control lines	0.166	0.194	0.016	0.138
Tajima’s D				
Longevity lines	−0.053	0.292	0.214	0.115
Control lines	−0.163	0.251	−0.201	0.011

We then ranked genes according to the increase in Tajima’s D-values in the longevity lines relative to control lines (Table 2). One of the top genes with differential D values was *intrepid* (*intr*) encoding a serine protease homolog, involved in negative regulation of female mating receptivity and sperm storage [35]. Other genes with differential D > 2 included *PH4alphaNE3* and CG31021, both involved in the oxidation-reduction process, *Prosbeta5R1*having endopeptidase activity, and *tailless* (*tll*), a transcription factor playing a role in a variety of developmental and metabolic processes. Overall, oxidoreductase and dioxygenase activities were the most significant GO term enrichments (*P* < 0.01) among genes with Tajima’s D values increased in the longevity lines (Additional file 1: Table S1). Among the top 200 genes with the largest difference in Tajima’s D, as many as 32 (16%, *P* = 0.058, Additional file 1: Table S1) were long non-coding RNAs (lncRNAs). In addition, a similar pattern of differentiation was found in eight small nucleolar RNAs and three miRNAs (mir-961, mir-968, and mir-2501, *P* = 0.018, Additional file 1: Table S1).

**Table 2:** List of top differentiated genes ranked according to the increase in Tajima’s D-values in the longevity lines relative to control lines

Gene Symbol	Diff_D	Selection_D	Control_D
CG42591	2.586	1.4701	−1.1159
snoRNA:Psi28S-3378	2.5005	1.5192	−0.9813
CR43358	2.4878	1.6261	−0.8617
CG43880	2.4578	1.1891	−1.2687
intr	2.4081	1.914	−0.4941
CG9168	2.3787	1.3942	−0.9845
Or94a	2.3183	1.9587	−0.3597
CR44225	2.305	1.3609	−0.9441
CR45322	2.3025	1.3841	−0.9184
CG34432	2.2633	1.6055	−0.6578
PH4alphaNE3	2.2457	1.594	−0.6518
CR44236	2.2203	1.2507	−0.9696
CG4763	2.1983	1.6423	−0.556
Prosbeta5R1	2.1706	1.5612	−0.6095
CG31021	2.137	1.52	−0.617
CG31093	2.1198	1.452	−0.6678
snoRNA:Psi18S-1389b	2.0825	1.2012	−0.8812
CG15398	2.0724	1.5682	−0.5042
tll	2.0553	1.0246	−1.0307
CR44713	2.0283	0.7839	−1.2444
CR44714	2.0283	0.7839	−1.2444
Or94b	2.0223	1.4939	−0.5284
CG32320	2.0082	0.818	−1.1902

### Simulations of neutral evolution and positive frequency-dependent selection

Since the longevity lines had an increasingly longer lifespan and the control lines thus had been through 94 more generations at the same time, it was necessary to control for the difference and determine a baseline (95% CIs) for heterozygosity and Tajima’s D changes in control lines due to drift effects alone. Simulations of neutral evolution under conditions mimicking our experimental system, based on genomic variation within the DGRP2, showed on average a 9.88% ± 0.02% (95% CIs) heterozygosity decrease in the group that went through ~2x more (201) generations than the other group (107) (Additional file 2: Figure S1). Drift effects on Tajima’s D after the 94 additional generations were negligible: −0.08% ± 0.61% (95% CIs) change. Separately, we also tested effects of positive frequency-dependent selection in conjunction with a bottleneck, which might enhance linkage disequilibrium (LD) and create selection for the commoner allele at the selected locus, potentially leading to an increase of heterozygosity in linked neutral loci. However, no increase of heterozygosity due to a stronger selection pressure was observed, compared with low/no selection under various recombination rates (Additional file 3: Figure S2).

### Signatures of positive selection

**Fig. 6:**
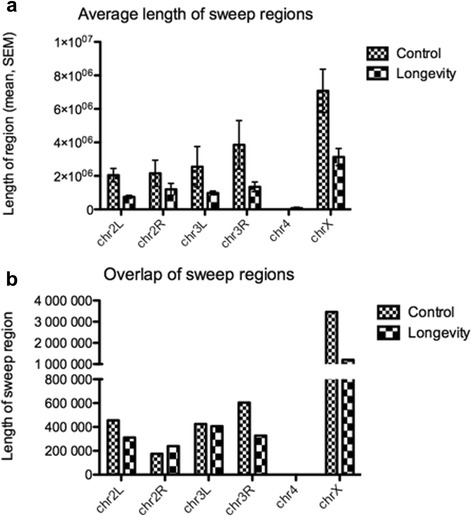
Sweeps found in the Longevity group and the Control group. Average length (±SEM) of sweep regions (**a**) and length of shared sweep regions (**b**) for Longevity and Control groups by chromosomes

Genomic regions of depleted genetic variation corresponding to putative selective sweeps were highly reduced in the selected lines (Fig. 6). One protein receptor gene (*Ptp10D*) belonging to the protein-tyrosine phosphatase family was under significant positive selection in the comparison between selection and control groups (Ka/Ks = 9, McDonald-Kreitman test *p* = 0.0217). *Ptp10D* is responsible for central nervous system development [36], axon guidance [37], and long-term memory [38]. Another gene with Ka/Ks = 4 (but MKT *p* > 0.05) was *Ank2*, involved in axon extension [39], neuron cellular homeostasis [39], sensory perception of pain [40] and short-term memory [41].

## Discussion

Strikingly, genome differentiation patterns produced by selection for increased lifespan are opposite to those we observed in selection for desiccation tolerance (Fig. 2b), evolving under strong positive selection [42]. Selection for longevity led to >20% genome-wide increase in heterozygosity relative to control lines, with coding sequences being proportionally more affected than other genomic regions. These results are inconsistent with neutral evolution, given the parallelism in responses across replicates within each group, and the experimental design controlling for numbers of flies used in each generation. Even if effective population sizes (N_e_) deviated from the experimental numbers of flies, N_e_ was presumably lower in selected lines, as some of the living old flies were likely past reproduction. Increased variance in reproductive output compared to young flies would lower N_e_ even further, which is in contrast to our observations. However, control lines went through approximately twice as many generations as selection lines at the same time, as a consequence of increasingly extended generation time in the selected group. Our simulations show that although this difference in generation numbers and associated drift effects accounted for approximately 50% of the observed decline in heterozygosity estimates for control lines, Tajima’s D estimates were essentially unaffected. Mutation accumulation effects alone are also unlikely to explain the pattern of variation, due to the moderate population sizes and generation numbers used in this experimental evolution system.

Balancing selection, which entails heterozygote advantage, negative frequency dependence or spatiotemporal heterogeneity (or a combination of any of these), thus emerges as the most likely mechanism capable of producing the increase in genetic diversity. By providing a means to maintain genetic variation, balancing selection is especially relevant to diversification of phenotypic variation in natural populations, including variation in life history traits, such as longevity. However, despite longstanding interest in the process of balancing selection, its prevalence, the extent of footprints in the genome, and evolutionary significance remain largely unknown due to challenges related to its detection [43–46]. Balancing selection is expected to boost neutral polymorphism in linked genome regions, in inverse proportion to their genetic map distances from the selected site, but the size and distribution of genomic regions showing indirect footprints of balancing selection have been unknown [45]. Our results prove that such effects at the genome-wide level can be pervasive and rapid, invoking newly established neutral polymorphisms from standing genetic variation.

Antagonistic pleiotropy theory of aging involves selection operating on genes with pleiotropic effects that can be subject to balancing selection [6], and some aging-related genes in *Drosophila*, such as *Ddc*, were in fact implicated in the evolution under balancing selection [47]. Life history traits in general evolve as a result of intricate trade-offs, or negative correlations, and antagonistic pleiotropy has been believed to play a central role in this process [6, 48, 49]. At the phenotypic level, there are two critical sources of correlations characterizing aging: the negative correlation between lifespan and reproduction [50–52], and the positive correlation between lifespan and stress tolerance [23, 53]. Antagonistic and non-antagonistic mechanisms of balancing selection are predicted to differentially affect population genomic signatures of recent balancing selection, with weaker signatures under antagonism [9]. Non-antagonistic processes entail partial selective sweeps that proceed rapidly, which results in stronger hitchhiking effects relative to partial sweeps from antagonistic selection [9]. The longevity lines exhibit very weak sweep signatures, even compared with the control lines, a pattern consistent with the antagonistic evolution.

However, even though antagonistic pleiotropy appears as a likely source of balancing selection accounting for the observed pattern of polymorphisms, the set of conditions under which this mechanism can be the sole driver of balancing selection without any temporal change in selection are limited [7–9]. Many insect species go through multiple generations per year, whereby different generations are exposed to different seasonal selection landscapes [16, 54], which is likely to result in a difference in optimal age of reproduction between cohorts. This change in selection landscape was mimicked in this study, as selection for longevity was relaxed every other generation, resulting in a substantial increase in longevity without a decrease in the reproductive capacity in early life [20]. We therefore argue that temporal shifts in selection coefficients on genes showing antagonistic pleiotropy for early life and late life fitness have driven maintenance of genetic variation in the selection lines while extending their longevity.

The association between stress resistance and lifespan has motivated the hypothesis that reactive oxygen species (ROS) cause aging [55] and led to experimental tests for lifespan extension by targeting activity of genes that promote antioxidant defenses. For example, overexpression of *Catalase* (*Cat*), *Superoxide dismutase* (*SOD*), *msrA*, and *glucose-6-phosphate dehydrogenase* (*G6PD*) all increased lifespan in *Drosophila* [56–59]. Even though we have not found significant polymorphism in these genes, genes with oxidoreductase activities that reduce or block oxygen in different forms from generating free-radical damage belonged to the most overrepresented group among those under the strongest balancing selection. Interestingly, many lncRNAs showed a polymorphism pattern consistent with signatures of balancing selection. Although their functions in *Drosophila* still await characterization, lncRNAs in mammals play important roles in a wide range of biological processes, including age-related diseases like cancer, cardiovascular pathologies, and neurodegenerative disorders [60]. In cucumber, some lncRNAs seem to be affected by balancing selection as well [61].

Drift and balancing selection are by no means the only evolutionary processes capable of increasing the levels of genetic diversity. Positive frequency-dependent selection, as selection for common alleles, is typically predicted to result in monomorphism, and therefore perceived as a process opposite to balancing selection. However, it occasionally may maintain rather than eliminate polymorphism, under the influence of their interactions with other alleles in the system [17]. One could argue that this mechanism may be sufficient to create hitchhiking effects on variability across the genome, especially when LD is increased due to bottlenecking of the experimental populations. Our simulations of this scenario under various LD (recombination) conditions and selection pressures (see Materials and Methods) failed to increase heterozygosity in the *in silico* longevity lines.

Genetic purging whereby allelic diversity is eroded by negative selection under inbreeding [62] potentially provides another alternative explanation of our results, assuming that purging of early-acting mildly deleterious variants in the control lines was more effective. However, the patterns of heterozygosity and Tajima’s D within recombination-suppressed regions are not consistent with purging effects. Since linkage slows down the decline of genetic diversity due to purging [18], one would expect the regions with suppressed recombination to produce the lowest difference in heterozygosity/diversity between longevity and control lines, a pattern opposite to actually observed. For example, the neighborhoods of centromeres representing regions of low recombination on the other chromosomes showed major increases in heterozygosity and Tajima’s D in the longevity lines relative to control lines. In another region characterized by a very low level of recombination, the ~71 Kb *yellow-achaete-scute* complex near the tip of chromosome X [63], the heterozygosity increase in the longevity lines was even more dramatic (2,281% compared with a 47% increase along the rest of the chromosome X). Detection of balanced polymorphism is enhanced by lower levels of recombination facilitating the correlation between genealogical histories of adjacent SNPs.

The *Drosophila* fourth chromosome is usually the smallest autosome (~5 Mb), with only a ~1 Mb euchromatic-like region of the right arm containing ~80 genes, believed to experience no—or very low—rates of recombination [64–68]. As predicted, chromosome 4 was most affected by selection for longevity and showed patters of increased heterozygosity in the selection lines. Notably, another study reported that the genetic variance in viability of *D. melanogaster* for the chromosome 4 was approximately one-half of that for the second chromosome [14], despite the fact that it contains less than 1/20th the number of genes. The chromosome is variable in several regions forming domains within 20-30% of the euchromatic arm with highly dimorphic haplotypes, already presumed to be maintained by balancing selection [66, 68].

Despite the paucity of sites with SNP polymorphism patterns that would suggest positive selection in the longevity lines, several genes still seem to have diverged between the selection and control groups through positive selection. The two most prominent were *Ptp10D* and *Ank2* with important roles in central nervous system and memory [36–39, 41].

## Conclusions

In sum, these results show that directional selection for extended life span in *D. melanogaster* leads to genetic diversification consistent with the operation of balancing selection either through antagonistic pleiotropy or cycling conditions between late reproduction and early reproduction generations. Such balancing selection effects may be prevalent in other ‘evolve-and-resequence’ experiments in which life history traits are under directional selection, but may go unnoticed when detection of genetic divergence and signatures of positive selection is the main focus of genomic analyses. Processes other than balancing selection, such as drift, genetic purging, and hitchhiking around sites under positive frequency-dependent selection (or their combination) may also be factors contributing to inflated genetic diversity, even if our simulations did not support such a conclusion in the presented case.

## Methods

### Drosophila culturing and experimental evolution

The lines were derived from a mass population of *D. melanogaster* established in our laboratory in September 2002. To ensure ample genetic diversity of the starting material, this population was founded by mixing four pre-existing laboratory stocks (600–700 flies from each). The stocks were discrete or mixed populations from four natural populations, two located in Denmark, one from Australia and one from the Netherlands, maintained in the laboratory in large numbers of breeding individuals. Flies were reared under low to moderately high density on standard *Drosophila* medium at 25 °C unless otherwise stated. The four stocks were:

1. Hov–Copenhagen basic strain

The flies were collected in two sites in Denmark (October 1997), Hvidovre (Zealand island, near Copenhagen) and Hov (Jutland peninsula, east coast). They were kept as 30 and 27 isofemale lines, respectively. The lines were mixed in February 1998 and maintained as one large interbreeding population.

2) Supermass Hov–Copenhagen population

This population was founded in September 2001 by mixing a number of heat-resistant and longevity selection lines. The heat-resistant selection lines were founded using offspring from the 16th generation of the Hov–Copenhagen basic strain. There were four sets of lines: lines selected for increased survival after heat shock (38.6 °C) with and without prior heat-hardening (37.0 °C), lines that were heat-hardened, but not selected, and lines reared at cycling temperatures (25 and 35 °C for 18 and 6 h, respectively). From May 2001, the first three sets were maintained without selection and/or hardening treatment. The longevity selection lines were established in April 2000 by sampling flies from the Hov– Copenhagen basic strain. They were selected for increased lifespan at two temperature regimes, 25 and 29 °C.

3) Heat-knockdown selection lines

These lines originate from two sets of highly inbred laboratory lines described by Norry et al. [69]. The first of them (SH) was founded by flies collected near Melbourne, Australia in February 1994 and selected for increased heat-knockdown resistance. The second set of lines (D) was founded by flies from the 10th generation of the Hov–Copenhagen basic strain and later selected for reduced heat-knockdown resistance.

4) Leiden strain

This strain was represented by 30 isofemale lines originating from females collected near Leiden (the Netherlands) in October 1999. For the first five generations it was maintained at 25 °C and then at 20 °C.

The mass population was maintained at 25 °C on a standard oatmeal-sugar-yeast-agar *Drosophila* medium. Every subsequent generation was founded using a mix of parents from different bottles. There were 25 bottles in total with ca. 50 pairs of parents per bottle. The six experimental lines described below (three selection and three control lines) were established by flies from the fourth generation of the mass population.

### Selection and control lines

Each replicate line was maintained in five culture bottles with a minimum population size of 60 pairs in each (in total a population size of 300 pairs). The five bottles, within a replicate line, were mixed each generation. The longevity selection took place every other generation. New emerged flies were placed in food vials and transferred to new vials every second day until approximately 50% mortality was reached. In the first generation of selection, this took 4 weeks, and after 48 generations of selection 50% mortality was reached after approximately 7–8 weeks. The surviving flies were used to start the next generation. Replicate lines of the control regime were allowed to breed within a week from eclosing and kept under standard laboratory conditions at 25°C and a 12/12 h light/dark cycle on standard agar–sugar–yeast–oatmeal medium. When flies for this experiment were sampled the lines selected for increased longevity had on average a 66% longer median lifespan than control lines in males and 63% in females [70, 71]. Flies from the selection group sampled for sequencing were offspring from an unselected generation, after 48 generations of selection (a total of 107 generations). Flies from the control regime used for sequencing were sampled after 201 generations. From each line 500 individuals of equal sex ratio were sampled. The DNA was extracted using the CTAB method in batches of 25 flies and pooled by line. TruSeq DNA libraries were prepared and sequenced on the HiSeq 2000 platform following Illumina’s protocols, and 2x90 bp paired-end reads were generated (Additional file 1: Table S2).

### Mapping and genotyping

The *Drosophila melanogaster* genome (dm3) and corresponding annotations (RefSeq) from UCSC (http://genome.ucsc.edu/) were used as reference for mapping. Raw reads were quality-controlled and filtered with FastqMcf [72]. The remaining reads were mapped to the reference using BWA [73] using default parameters. GATK [74] with default parameters (except for using ‘--sample_ploidy’ for pooled data and setting –heterozygosity to 0.01) was employed to generate genotypes in each line. Genotypes with more than 2 alleles were discarded. Only sites with genotyping quality greater than 30, minimum depth 10, and maximum depth 250 were used in the analysis.

### Estimates of Fst, π, θ, Tajima’s D, and heterozygosity

Samtools [75] was used to generate the pileup result. SNPs within 10 bp of indels were discarded. F_ST_ value for each SNP was generated using Poopolation2 [76], while PoPoolation [77] was used to calculate π, Watterson's θ and Tajima’s D with the window size set to 10 Kb. Heterozygosity was estimated using a 100 Kb sliding window with a step of 10 Kb.

### Sweep region detection

Putative selective sweep regions were detected with Pool-hmm [78], a hidden Markov model for finding selective sweep signatures from Pool-Seq data. The parameters used in Pool-hmm were “-n 100 -c 5 -C 400 -q 20 -e sanger -p -k 0.0000000001”, while “--theta” was set to be the *θ* estimated for each sample.

### Neutrality and positive frequency-dependent selection simulations

Genome simulations under a neutrality model were conducted using forqs [79]. The haplotype data for the simulation were obtained from the Drosophila Genetic Reference Panel 2 (DGRP2, http://dgrp2.gnets.ncsu.edu/) of 205 inbreed lines. The mass-breeding phase of the experiment was simulated for 1,000 generations with a population size of 100,000. Conditions corresponding to our experimental system were simulated for 201 generations with population size limited to 300 individuals. Two types of simulations were performed: 1) To test whether observed patterns of genomic differentiation could be produced by drift alone, the recombination rate was set to 2 and no selection was added. Simulations were run three times to mimic the replicas in our study. A total of 6 chromosomes/arms (chr2L, chr2R, chr3L, chr3R, chr4 and chrX) were generated. Missing genotypes were set as heterozygous. Heterozygosity and Tajima’s D were computed based on the same window size as for the real experimental data and compared between generations 107 and 201, corresponding to the experimental gap between longevity and control lines, respectively. 2) To test the hitchhiking effects of positive frequency-dependent selection in conjunction with increased LD, the recombination rate was set to either 2 or 0.2 for testing under conditions of moderate and low recombination. A 1.1M-size region from chr2L:9,500,000-11,500,000 was simulated, while a total of 22 selected sites were evenly distributed along the region. The initial deleterious allele frequencies for all selected sites were set to 0.1 (under the Hardy-Weinberg equilibrium) and fitness for the deleterious allele was set to 0.9 or 0.999 for mimicking strong selection or weak/no selection. A total of 1,000 generations were simulated and the simulations were repeated 100 times. Heterozygosity was calculated with the same window size (100 Kb) along this region every 100 generations.

## Additional files

Additional file 1: Table S1. Summary of genes with the largest differential Tajima’s D values. **Table S2**. Summary of analyzed reads. (XLSX 32 kb)
Additional file 2: Figure S1. Simulations of heterozygosity decrease under neutral evolution. (PNG 152 kb)
Additional file 3: Figure S2. Simulations of positive frequency-dependent selection pressures (strong or weak) under moderate (A) or low (B) recombination rates. (TIFF 5741 kb)
