# Supplementary figures and images for: Nucleotide diversity inflation as a genome-wide response to experimental lifespan extension in Drosophila melanogaster
Source: BMC Genomics. 2017 Jan 14;18:84. doi: 10.1186/s12864-017-3485-0 (PMC5237518; doi:10.1186/s12864-017-3485-0)

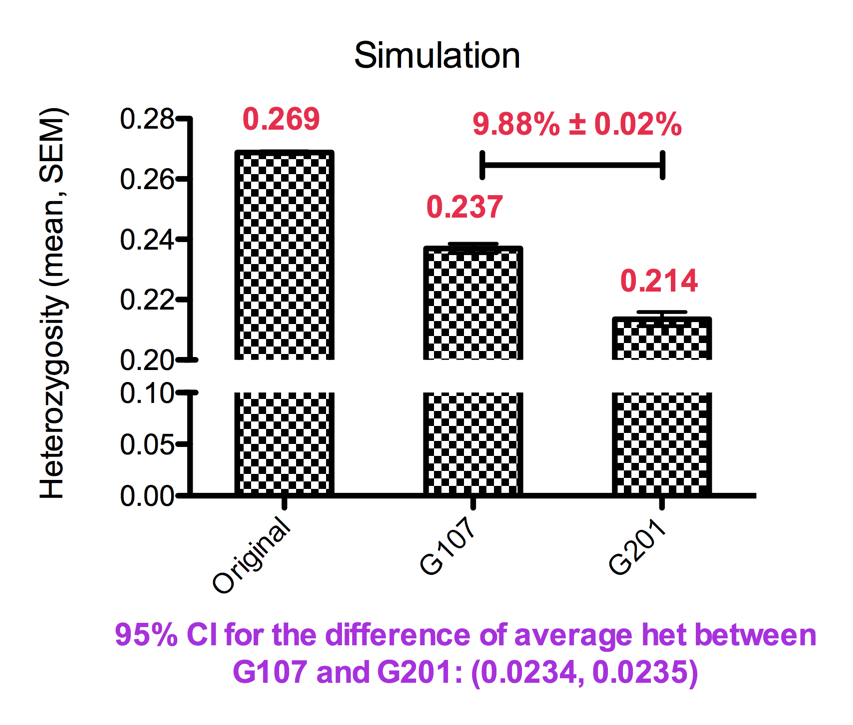

Supplement: Additional file 2: Figure S1. — Simulations of heterozygosity decrease under neutral evolution. (PNG 152 kb) [file 12864_2017_3485_MOESM2_ESM.png]

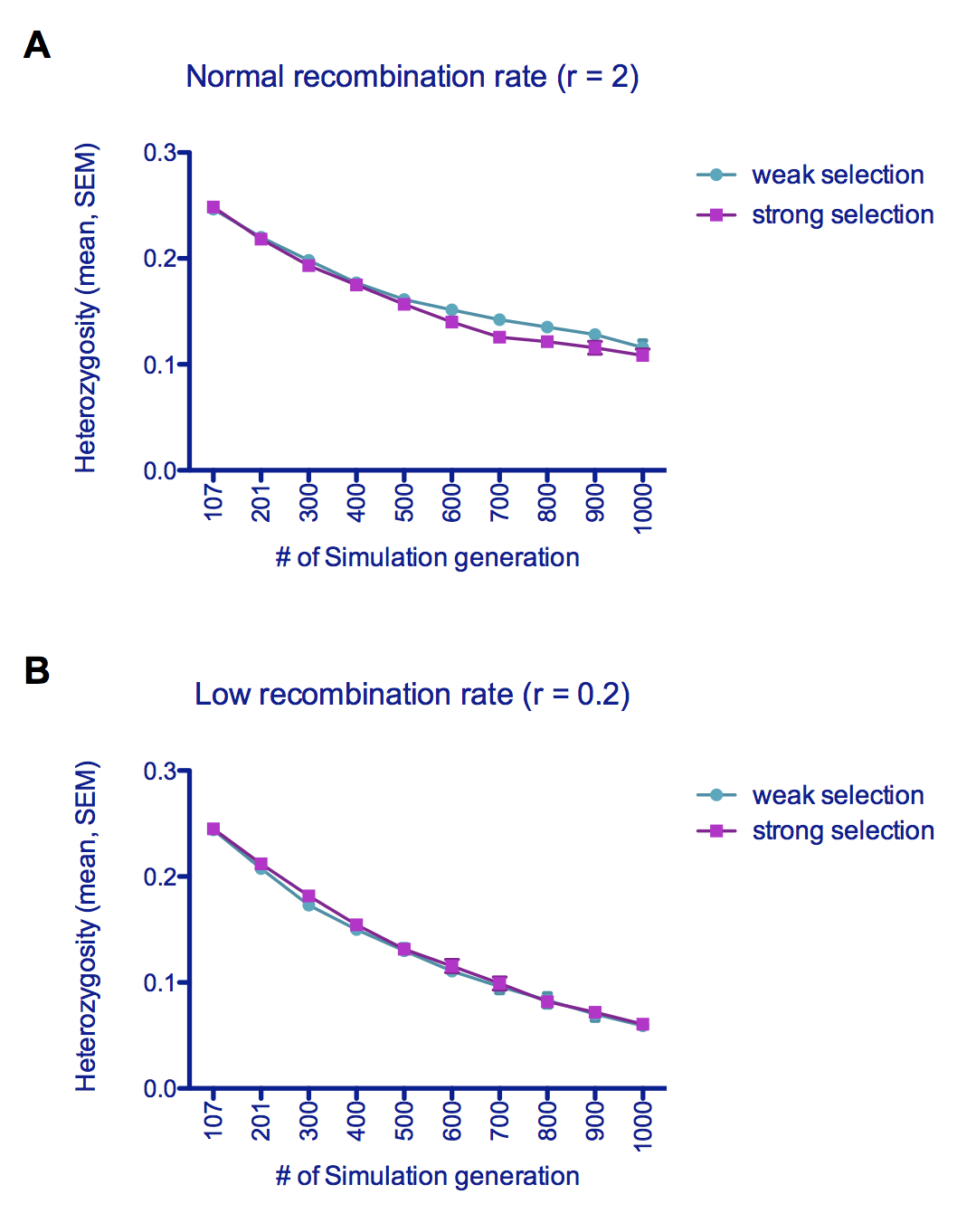

Supplement: Additional file 3: Figure S2. — Simulations of positive frequency-dependent selection pressures (strong or weak) under moderate (A) or low (B) recombination rates. (TIFF 5741 kb) [file 12864_2017_3485_MOESM3_ESM.tiff]
